# A Severe Manic Episode Induced by Corticosteroid Treatment in a Patient With Subthreshold Bipolar Disorder

**DOI:** 10.7759/cureus.78765

**Published:** 2025-02-09

**Authors:** Tomonori Hara, Yusuke Kaichi, Keisuke Imai, Shiho Hattori

**Affiliations:** 1 Psychiatry, Hatsuishi Hospital, Kashiwa, JPN

**Keywords:** aripiprazole, asthma, bipolar disorder, coritosteroid-induced mania, olanzapine, steroid-induced mania

## Abstract

Corticosteroid treatment sometimes causes psychiatric side effects such as mania, depression, and psychosis. It is believed that exogenous corticosteroids lead to dysregulation of corticosteroid signaling and neurotransmitters in a dose-dependent manner. Therefore, the administration of corticosteroids is at risk of worsening bipolar disorder. Here, we present a case of a female patient with subthreshold untreated bipolar disorder who experienced a severe manic episode after corticosteroid treatment. She had a history of childhood asthma but no remarkable psychiatric medical history except for subthreshold mood swings. At age 30, she had her first asthma attack in over 10 years, for which she received intravenous corticosteroids. A week later she was admitted to a psychiatric hospital due to severe manic symptoms. She was diagnosed with corticosteroid-induced mania and was treated with olanzapine. After remission, olanzapine was gradually reduced. However, she experienced a recurrent manic episode. Her diagnosis was updated to bipolar disorder, and she has resumed medication. This case highlights the risk of corticosteroids worsening bipolar disorder and the need to carefully assess previous psychiatric symptoms before using corticosteroids.

## Introduction

Corticosteroids are broadly classified into glucocorticoids and mineralocorticoids, which play a role in maintaining homeostasis in the organism. They have anti-inflammatory and immunosuppressive effects; therefore, they are used as powerful treatments for inflammatory or autoimmune diseases such as asthma or rheumatoid arthritis [1]. Meanwhile, they have side effects, including psychiatric symptoms known as steroid-induced psychosis, which are abnormal mental states caused by exogenous corticosteroids. The risk is dose-dependent. Previous studies have found that psychiatric symptoms were present in 1.3%, 4.6%, and 18.4% of patients taking < 40 mg/day, 41-80 mg/day, and > 80 mg/day of prednisone, one type of corticosteroid, respectively [2,3]. Among psychiatric symptoms, manic state accounted for 35 %, depressive state for 28%, and psychotic state for 11% [4]. These symptoms typically appear on the third or fourth day after starting treatment, but they can occur at any time, even after the treatment has ended [5].

The pathophysiology of corticosteroid-induced psychiatric symptoms is not fully understood. However, it is believed to involve dysregulation of the hypothalamic-pituitary-adrenal axis and dysregulation of neurotransmitters, chemical messengers in the nervous system, including dopamine and 5-hydroxytryptamine (5-HT) whose alterations are implicated in bipolar disorder, schizophrenia, and major depressive disorder [5-8].

The diagnosis heavily relies on the patient's history of corticosteroid use. It can be complicated when there are psychiatric comorbidities, as the use of corticosteroids can affect existing psychiatric symptoms. Here, we report a case with a severe manic episode induced by corticosteroid treatment in a patient with subthreshold, untreated bipolar disorder.

## Case presentation

A 30-year-old woman had a history of childhood asthma, but her last attack was around age 10. The follow-up had been discontinued over 10 years ago. She also had mild mood swings since adolescence, but they were not severe enough to see a psychiatrist. After high school, she held various jobs, including in the amusement industry. Upon her hospitalization, she was temporarily unemployed but willing to work. Her family history is unremarkable. At age 30, she experienced an asthma attack after a long interval. Her peripheral oxygen saturation (SpO2) was dropped to 85 % on room air. She was admitted to a hospital and received 160 mg/day of methylprednisolone sodium succinate intravenously for five days. After eight days in the hospital, she was discharged.

On the third day post-discharge, she experienced an elevated mood with talkativeness, ideation, decreased need for sleep, grandiosity, increase in goal-directed activity, and psychomotor agitation. She unnecessarily went to the police station to complain about trivial matters. At home, she loudly sang songs, took off her clothes, and ran out to the balcony. When she arrived at the hospital, she was fully conscious but exhibited elevated mood and psychomotor excitement. She shouted nonsensical things in a talkative and aggressive manner, frightening everyone around her. She attempted to take off her clothes. She denied experiencing any hallucinations, including auditory ones, and denied having delusions. There were no signs of cognitive dysfunction. SIGNIFY ER Drug Screen Test (Abbot, IL, USA) was all negative for phencyclidine, benzodiazepines, cocaine metabolites, amphetamines, cannabinoids, opiates, barbiturates, tricyclic antidepressants, methylenedioxymethamphetamine, oxycodone, and propoxyphene. She was involuntarily admitted to the hospital on the seventh day post-discharge. Her Young Mania Rating Scale (YMRS) score [9] upon admission was 41. Due to the severity of her psychomotor agitation, olanzapine 10 mg was administered intramuscularly on the first day of hospitalization. From the second day, she took olanzapine orally. The results of blood tests, head CT scans, and electroencephalography were unremarkable. Her symptoms were compatible with the criteria of the manic episode in the Diagnostic and Statistical Manual of Mental Disorders (DSM)-5. Throughout the period of hospitalization, inhaled corticosteroids and β2 stimulants were continued to prevent asthma attacks (budesonide 160 μg and formoterol fumarate hydrate 4.5 μg, twice daily). Her manic symptoms gradually improved after increasing her olanzapine dosage to 20 mg/day. After reaching remission (YMRS score: 0), her cognitive functioning was assessed with the Wechsler Adult Intelligence Scale Fourth Edition (WAIS-IV). Her full-scale intelligence quotient (IQ), verbal comprehension index, perceptual reasoning Index, working memory index, and processing speed index were 108, 113, 118, 100, and 85, respectively.

Five weeks after completion of the 160 mg/day of methylprednisolone, olanzapine was tapered because the adverse effect of corticosteroid was likely to resolve by six weeks in most cases [5]. Hyperprolactinemia (serum prolactin: 55.0 ng/ml) and subsequent amenorrhea, possibly related to the high dose of olanzapine, were also reasons for tapering. Because her menstrual cycle had been regular, the prolactin level was measured for the first time. As of the following week, the dosage of olanzapine was 10 mg/day. A few days after reducing olanzapine to 2.5 mg/day (eight weeks after admission), she experienced a recurrent manic episode with elevated mood, talkativeness, ideation, decreased need for sleep, and increased goal-directed activity (YMRS score: 20). The diagnosis was updated to bipolar disorder considering her history of subthreshold mood swings and the timing of the improvement in corticosteroid side effects estimated by literature [5]. Olanzapine was switched to aripiprazole due to hyperprolactinemia. Her manic episode gradually improved after the start of aripiprazole. Serum prolactin levels decreased to 5.3 ng/ml. Aripiprazole was gradually reduced from 24 mg to 15 mg/day because of extrapyramidal symptoms. Though there was slight mood instability, no apparent relapse was observed afterward (YMRS score: 0). She was discharged after a four-month stay at the hospital and continued to receive outpatient treatment.

## Discussion

We have presented a patient with subthreshold bipolar disorder who experienced a severe manic episode after intravenous corticosteroid treatment. Although there are several reports of bipolar disorder worsening with corticosteroids [10-13], this is the first report of severe exacerbation that occurred in an unrecognized, subclinical case of bipolar disorder.

It is crucial to determine if exacerbation stems from the original psychiatric illness or is independently triggered by corticosteroids in patients with psychiatric comorbidities. The initiation of corticosteroids or an increase in dosage usually occurs in the background of a stressful physical issue. This stress can be a risk for worsening, such as schizophrenia, bipolar disorder, or major depressive disorder, making it challenging to fully distinguish between these pathologies. In our case, given that she had suffered from asthma since childhood and had been hospitalized for it in the past, we concluded that her manic episode was more likely induced by corticosteroids rather than being a result of stress from her physical illness and hospitalization.

In this case, inhaled corticosteroids and β2 stimulants were continued to prevent another asthma attack. Although they can induce psychiatric symptoms as previously reported, they were continued because they have fewer systemic side effects than the same dose of oral corticosteroids and their doses are not high (160 μg budesonide is equivalent to 2.1 mg prednisone; 160 mg methylprednisolone used for her is equivalent to 200 mg prednisone) [14,15]. Given the dose of corticosteroids, the estimated probability of her experiencing psychiatric symptoms was 18.4% [2,3]. However, the risk was likely higher because she received corticosteroids intravenously, which could potentially increase the concentration of corticosteroids in her blood compared to oral administration.

The first-line treatment for corticosteroid-induced psychiatric symptoms is tapering or discontinuation of corticosteroid treatment [16]. In addition, risperidone, olanzapine, and quetiapine are known to be effective for corticosteroid-induced mania [17-19]. In our case, olanzapine was effective in the acute treatment of a corticosteroid-induced manic episode. However, we started the administration of olanzapine prior to changing her diagnosis from a single manic episode induced by corticosteroid to bipolar disorder, which was effective. Olanzapine may be useful in acute treatment when the diagnosis is uncertain between bipolar disorder and corticosteroid-induced psychiatric symptoms.

## Conclusions

This case report describes a patient with subclinical bipolar disorder who experienced a severe manic episode induced by intravenous methylprednisolone. This report underscores the risk that corticosteroids can exacerbate bipolar disorder and the need for careful evaluation of past psychiatric symptoms before corticosteroid use. Even in the absence of a history of psychiatric attendance, subthreshold psychiatric symptoms should be fully considered to optimize the risk of corticosteroid treatment.
